# Immunohistochemical Analysis of pH-Sensitive TRPV5 in Common Skin Tumors

**DOI:** 10.3390/ijms27125287

**Published:** 2026-06-11

**Authors:** Sophia Victoria Weiß, Judith Heider, Dennis Niebel, Katja Evert, Florian Zeman, Marietta von Süßkind-Schwendi, Daniel Schiltz, Tobias Ettl, Christoph Brochhausen, Stephan Schreml

**Affiliations:** 1 Department of Dermatology, University Medical Center Regensburg, Franz-Josef-Strauß-Allee 11, 93053 Regensburg, Germany; sophia.v.weiss@gmail.com (S.V.W.); judith.heider@ukr.de (J.H.); dennis.niebel@med.uni-regensburg.de (D.N.); 2 Institute of Pathology, University of Regensburg, Franz-Josef-Strauß-Allee 11, 93053 Regensburg, Germany; katja.evert@klinik.uni-regensburg.de; 3 Center for Clinical Studies, University Medical Center Regensburg, Franz-Josef-Strauß-Allee 11, 93053 Regensburg, Germany; florian.zeman@ukr.de; 4 Institute for Physiology and Biochemistry of Diet, Max Rubner-Institute, Bundesforschungsinstitut für Ernährung und Lebensmittel, Haid-und-Neu-Straße 9, 76131 Karlsruhe, Germany; marietta.vonsuesskind-schwendi@mri.bund.de; 5 Klinik für Plastische und Ästhetische Chirurgie, Handchirurgie, Helios Klinikum Emil von Behring, 14163 Berlin, Germany; daniel.schiltz@helios-gesundheit.de; 6 Department of Maxillofacial Surgery, University Medical Center Regensburg, Franz-Josef-Strauß-Allee 11, 93053 Regensburg, Germany; tobias.ettl@ukr.de; 7 Institute of Pathology, University Medical Center Mannheim, Medical Faculty Mannheim, Heidelberg University, Theodor-Kutzer-Ufer 1-3, 68167 Mannheim, Germany; christoph.brochhausen-delius@umm.de

**Keywords:** TRPV5, melanoma, squamous cell carcinoma, basal cell carcinoma, tumor, microenvironment, pH

## Abstract

Transient receptor potential vanilloid 5 (TRPV5) is a calcium- and pH-sensitive ion channel. It plays a role in tumor biology and cellular calcium homeostasis. Due to the inverse pH gradient in solid tumors (extracellular acidosis and increased intracellular pH), TRPV5 is interesting as a signaling molecule in tumors, as the altered pH in the tumor microenvironment (TME) impacts tumor growth and metastasis. This is the first study to analyze the expression of TRPV5 in common skin cancers, i.e., basal cell carcinomas (BCC), squamous cell carcinomas (SCC), malignant melanomas (MM) and melanocytic nevi (MCN). The results showed a significantly lower expression of TRPV5 in BCC than in all other tumor entities analyzed. While less than half of the BCC were positive for TRPV5, SCC, MM, and MCN exhibited a high level of positive staining results. These results suggest that TRPV5 may especially help as a novel marker in the differentiation of SCC from BCC. The low expression of TRPV5 in BCC, a rarely metastatic tumor, may also point to a role of TRPV5 in the progression of epithelial skin tumors. Further functional studies, however, are needed to clarify the exact role of TRPV5 in skin tumors.

## 1. Introduction

The worldwide prevalence of skin cancer has been rising for decades. An extensive examination of the Global Burden of Disease Study revealed that the age-standardized incidence rate for skin cancer rose by 1.94% per year from 1990 to 2021 [1]. The most common types are non-melanoma skin cancers (NMSC), such as SCC and BCC. For instance, between 1990 and 2017, the incidence of SCC increased by 310% [2]. The rapidly rising rates of MM are particularly problematic. In the USA, the number of new cases has tripled in the last 40 years. This increase can be partially explained by demographic change, better diagnostics, and greater UV exposure [1]. Despite constituting about 1% of all skin cancers, MM accounts for over 80% of skin cancer-related fatalities owing to its propensity to metastasize [3]. Although SCC is a non-melanocytic form of skin cancer, it presents a markedly elevated risk of metastasis and mortality when compared to BCC, particularly in advanced tumors [4]. No intervention is generally required for MCN, which are benign melanocytic lesions. Nonetheless, MMs may result from MCN [5], even though this concept is still debated.

The physiological tissue microenvironment (TME) is disrupted in solid tumors, such as skin tumors. An inverted pH gradient is established, i.e., the extracellular pH (pHe) is lower than the intracellular pH (pHi), resulting in pHe < pHi (inside-out pH gradient, inverse pH gradient) [6,7]. This inverse pH gradient is a hallmark of numerous solid cancers and, in addition to its effect on cellular proliferation, it also affects invasiveness and tumor metabolism [6,7]. The acidic pH of tumors results partially from heightened lactate metabolism (Warburg effect) and inadequate blood supply (hypoxia), and it is believed to facilitate metastasis and invasive growth [8]. Apart from that, the inverse pH gradient is controlled via a plethora of proteins, such as NHE1, MCTs, carbonic anhydrases, and VATPase [9]. The reverse pH gradient also leads to the death of healthy cells, which require a higher extracellular pH of >7.2.

Transient receptor potential (TRP) channels participate in cell migration, cell proliferation, invasion and angiogenesis, all of which are associated with cancer progression. TRPV5 is one of the two channels within the TRPV group that shows a strong selectivity for calcium ions [10] and is pH-sensitive. Within the cell, TRPV5 is localized in intracellular vesicles and in the plasma membrane. Depending on the pHe, the channel can be translocated from intracellular vesicles to the membrane, also allowing dynamic regulation of calcium influx. TRPV5 has also been detected near the nucleus [11,12]. It is regulated by hormones like 1,25-dihydroxyvitamin D_3_, parathyroid hormone, estrogen, and testosterone. The activity of TRPV5 is also influenced by factors such as plasmin, protons (pHe), magnesium, and calcium [13,14]. The influx of calcium into the cells via the TRPV5 Ca^2+^ channel elevates intracellular calcium concentration, which subsequently inhibits the activity of TRPV5 via negative feedback. Hence, TRPV5 is involved in calcium homeostasis of tumors, and a potential interplay with tumor metabolism has been suggested [15].

Furthermore, TRPV5 basal channel activity is regulated via activation by phosphatidylinositol-4,5-bisphosphate (PI(4,5)P2) and inhibition by Ca^2+^-bound calmodulin (CaM), while parathyroid hormone (PTH) increases the activity of TRPV5 via protein kinase A (PKA)-mediated phosphorylation. Low pH, e.g., in the tumor microenvironment (TME), leads to reduced TRPV5 activity by preventing PI(4,5)P2-activation. Additionally, low pH causes TRPV5 to go from an open to a closed state. TRPV5 is therefore modulated by two key extrinsic factors: low pHe and PKA [16]. Furthermore, it has been shown that TRPV5 regulates immune cell (i.e., B cell) activation and signaling via PI3K-Rhoa pathways [17], a mechanism very important for current immunomodulatory therapies.

Recent studies in acute, chronic and radiogenic wounds provide further insight into the possible physiological significance of pHe and its interplay with the repair process [18]. Wound repair shares many similarities with tumor growth, including proliferation and migration [19,20]. Considering TRPV5’s pronounced pH-sensitivity, these alterations within the wound microenvironment could potentially regulate TRPV5-mediated calcium influx within keratinocytes and endothelial cells. Consequently, this modulation may impact essential processes in wound repair as well.

However, TRPV5 is not the sole receptor that is affected by variation in pH levels. While a reduced pHe inhibits calcium uptake via TRPV5 [21], a reduced pHe also modulates the activity of various receptors that are sensitive to protons, e.g., certain G protein-coupled receptors (GPCR, GPR4/19/31/65/68/132/151), acid-sensitive ion channels (ASIC), other transient receptor potential vanilloid channels (TRPV), TWIK-related acid-sensitive potassium channels (TASK) and transient receptor potential canonical channels (TRPC). Initial data regarding the expression profiles of pH-sensitive TRPC, GPCR, TASK/TRPV, and ASIC in different skin tumors have recently been published [22] and show that BCC tend to exhibit significantly lower levels of these pH-sensitive proteins compared to SCC, MCN and MM. In addition, it has been demonstrated that targeted manipulation of calcium channels can trigger calcium-dependent stress in the endoplasmic reticulum, which in turn influences drug resistance [23,24]. The targeted regulation of calcium channels, such as TRPV5, has been discussed as a potential therapeutic approach, even though its applicability in patients with metastatic tumors still needs to be investigated [25]. Given that knowledge about the interplay between TRPV5 and skin tumors is currently very limited, it is crucial to expand research in this area. To our knowledge, the present study is the first to examine the expression of pH-sensitive TRPV5 in the above-mentioned skin tumors.

## 2. Results

### 2.1. Positive and Negative Controls

**Figure 1:**
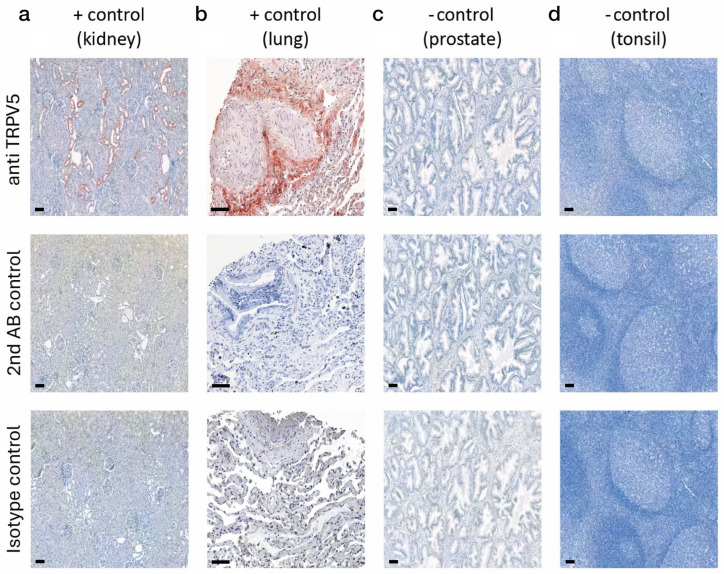
Tissue controls for the immunohistochemical staining of TRPV5. (**a**,**b**) Kidney and lung were used as positive controls for TRPV5 and showed positive staining compared to conditions without primary antibody and isoantibody control. (**c**,**d**) Prostate and tonsil were used as negative controls for TRPV5. No reaction was observed in either the positive control or the isoantibody control. Scale bar represents 200 µm.

Negative controls were implemented using isotype-matched control antibodies and by removing the primary antibody (see Section 4 for protocol). Kidney and lung tissues served as positive controls for validation (Figure 1a,b), whereas prostate and tonsil tissue were used as negative controls (Figure 1c,d).

### 2.2. Western Blots

**Figure 2:**
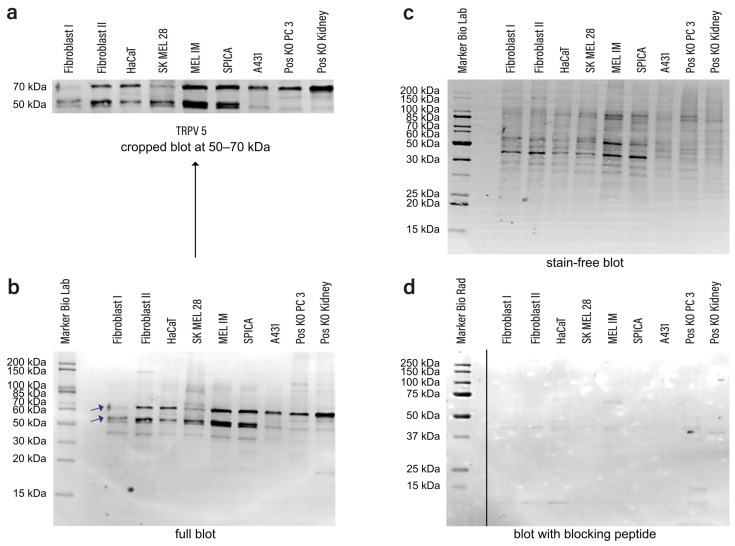
Western blots for TRPV5. Assessment of the specificity of the TRPV5 antibody via Western blots at a dose of 20 μg (=10 µL) per lane in various cell lines. Gel: any kD TGX stain-free precast gel. Normalization was conducted based on the total protein content of each lane, rather than utilizing a specific housekeeping protein, as facilitated by the TGX stain-free gel system, in comparison to the markers from BioLab and BioRad, respectively. (**a**) Cropped version of the full Western blot in (**b**). 2 bands at ~70 kDa and ~50 kDa, as expected from the datasheet. Correction for minor slant in gel photograph of (**b**). (**b**) Full blot with entire lanes of (**a**). Arrows point to the bands shown in (**a**). (**c**) Stain-free blot after separation of the proteins and blotting. (**d**) Blot with blocking peptide. To verify the specificity of the anti-TRPV5 antibody, preincubation with the corresponding blocking peptide was conducted, which resulted in a total loss of signal. Only in the control lane without blocking peptide are the respective bands visible.

Using Western blots, the selectivity of the previously mentioned primary antibody TRPV5 was confirmed (Figure 2a–d; original green fluorescent and uncropped versions of Figure 2b,d are shown in Supplementary Figure S1). At around 70 kDa, bands were detected. According to the manufacturer’s data sheet, the predicted molecular weight of TRPV5 is roughly 75 kDa. The second slightly lower band is most likely due to partial proteolysis. The Western blot was repeated a total of five times and additionally performed once with the control peptide. The data show successful detection of TRPV5 and selectivity of the antibody used in this assay.

### 2.3. Tissue Samples

Initially, 25 tumor samples were chosen for each tumor type. In certain instances, the remaining tissue in the chosen paraffin blocks was insufficient for performing all immunohistochemical staining. Furthermore, technical issues, including overstaining or insufficient preservation of tumor cell nests in the tissue sections, rendered certain samples unusable for evaluation. For BCC, nodular, superficial and mixed forms (partially nodular and sclerosing) were analyzed (2 of 24 superficial, 1 of 24 mixed and 21 of 24 nodular). Regarding SCC, all analyzed samples were invasive, as non-invasive forms would be classified as actinic keratoses. All MMs were superficial spreading with occasional secondary nodular components. As a result, the final count of cases differed among the various tumor types as follows: BCC n = 24, SCC n = 21, MCN n = 20, and MM n = 22. As BCC and SCC are epithelial tumors that originate from the epidermis, in the evaluation no distinction between epidermal and dermal portions was made, as both are connected per definitionem (except for metastases, which were not part of this study).

### 2.4. Immunohistochemistry

Figure 3 illustrates representative staining results along with their respective scorings. A detailed list of all staining and scoring results can be found in Supplementary Figures S2–S9 and Supplementary Tables S1–S4. The assessment system is explained and detailed in the Methods Section about scoring.

**Figure 3:**
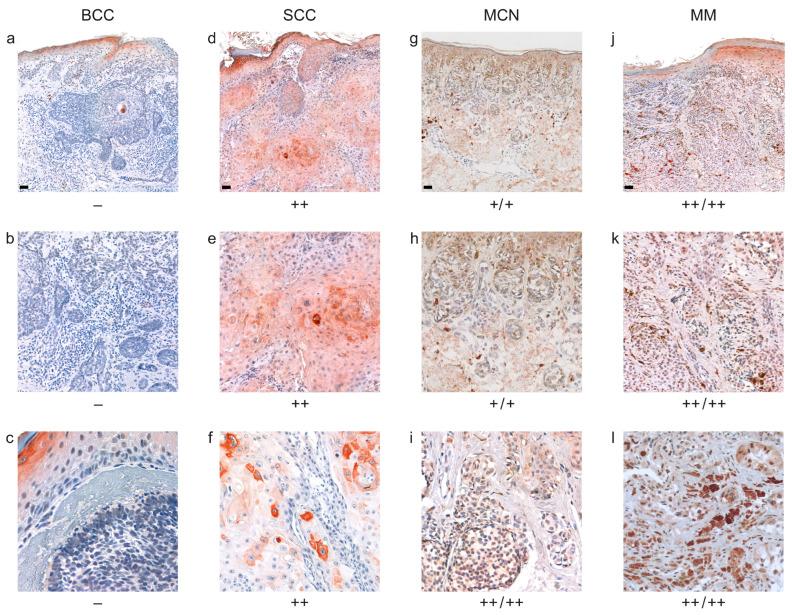
Immunohistochemical staining for TRPV5 in BCC, SCC, MCN and MM. Staining was classified into the following categories: ++ for strong positive responses, + for weak-positive or partially positive responses, and − for negative responses. For details, see Section 4. As shown in (**a**–**c**), BCC exhibit a very low expression of TRPV5 (**a**,**b** = patient 20, **c** = patient 21, Supplementary Figure S3 and Table S1). All other BCC are shown in Supplementary Figures S2 and S3. SCC mainly show a strong positive reaction (**d**,**e**: patient 4, **f** = patient 5, Supplementary Figure S4 and Table S2). All additional SCC are shown in Supplementary Figures S4 and S5. The dermal and epidermal portions of MCN (**g**,**h** = patient 18, **i** = patient 8, Supplementary Figure S7 and Table S3) exhibit a positive reaction for TRPV5 (all other MCN in Supplementary Figures S6 and S7). The epidermal and dermal portions of MM (**j**,**k** = patient 4, **l** = patient 15, Supplementary Figure S8 and Table S4) mostly exhibit positive expression of TRPV5 as well (all cases in Supplementary Figures S8 and S9). Results are summarized in Figure 4. Scale bars represent 200 µm. Increasing magnification of 10× (**a**,**d**,**g**,**j**); 20× (**b**,**e**,**h**,**k**); and 40× (**c**,**f**,**i**,**l**) were used for better visualization. We refer to Supplementary Figures S2–S9 and Supplementary Tables S1–S4 for all other staining results.

**Figure 4:**
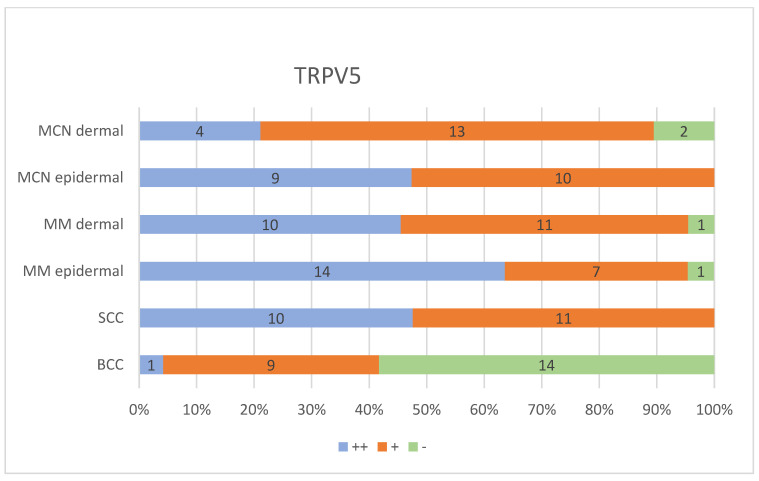
Summary of immunohistochemical scoring results. In short, scoring was done as follows: ++ for strong positive responses, + for weak-positive or partially positive responses, and − for negative responses. For details, see Section 4. Staining of the epidermis served as an internal reference for each respective case. For details, see Section 4. The bars represent the occurrence of the particular score. Only the scorings for MM/MCN were separated into dermal and epidermal portions, as BCC/SCC are epithelial tumors that originate from the epidermis and are connected with it. For individual scores, see Supplementary Tables S1–S5.

### 2.5. Basal Cell Carcinomas (BCC)

A total of 37.5% of the cases showed weak positive staining. In 58.3%, a negative reaction for TRPV5 was detected. Only 1 out of 24 (4.2%) was classified as strong (Figure 3, Supplementary Figures S2 and S3, Supplementary Tables S1 and S5).

### 2.6. Squamous Cell Carcinomas (SCC)

All 21 samples showed either a slight or strong positive reaction. An amount of 11 of 21, or 52.4%, showed a weak positive staining. 47.6% (10 of 21) were categorized as strong positive (Figure 3, Supplementary Figures S4 and S5, Supplementary Tables S2 and S5).

### 2.7. Melanocytic Cell Nevi (MCN)

The examination of the samples for MCN was split into epidermal and dermal sections. In the epidermal section, 52.6% (10 of 19) exhibited weak positive staining, whereas 47.4% (9 of 19) demonstrated strong positive staining. In the dermal sections, 2 of 19 (10.5%) were classified as exhibiting a negative reaction, 13 of 19 (68.4%) displayed moderate positive staining, and 4 of 19 (21.1%) were assessed as strong positive. To separate it more accurately, 7 of 19 appeared to be weakly positive for TRPV5 in the epidermal as well as in the dermal part. Four specimens showed strong positive staining in both epidermal and dermal sections, and only two samples were categorized as moderately positive in the epidermal part and negative in the dermal section (Figure 3, Supplementary Figures S6 and S7, Supplementary Tables S3 and S5).

### 2.8. Malignant Melanomas (MM)

For MM, 10 of 22 were rated as strongly positive in the dermal and epidermal parts, 6 samples exhibited weak positive staining in both sections and 4 histological specimens appeared strongly positive in the epidermal portion and moderately positive in the dermal sections. Only 2 of 22 demonstrated a negative reaction in one layer and weak positive staining for the other tissue area. In the epidermal portion, 4.5% were negative, 31.8% showed moderately positive staining and 63.6% were found to be strongly positive. Evaluating the dermal parts, 4.5% were negative, 50% were weakly positive and 45.5% showed strong positive staining (Figure 3, Supplementary Figures S8 and S9, Supplementary Tables S4 and S5). Ulceration was present in only 1 out of 22 MM cases (case no. 3), with no difference in the overall expression pattern as compared to the other MM.

### 2.9. Results of Statistical Analysis

TRPV5 expression was significantly lower in BCC in comparison to SCC (adj. *p* < 0.001), as well as between BCC and epidermal portions of MCN (adj. *p* < 0.001). In the comparison of dermal proportions of MCN with BCC, a trend towards a difference could be observed, but no statistical significance was achieved after correction (adj. *p* < 0.097). The amount of positive staining for TRPV5 was also considerably lower in BCC than in epidermal (adj. *p* < 0.001) and dermal sections of MM (adj. *p* < 0.001). No significant variation in TRPV5 expression was identified between MCN and MM (dermal and epidermal), dermal/epidermal portions of MCN and SCC or SCC and dermal/epidermal parts of MM (Table 1 and Supplementary Table S5).

Inter-rater reliability was very high for all comparable samples (n = 127; 1 NCN only had an epidermal portion, 1 NCN only had a dermal portion) with a Cohen’s Kappa of 0.948 (95% CI: 0.907–0.989) and a Krippendorff’s alpha of 0.952 (95% CI: 0.905–0.985). Minor discrepancies were only seen in the evaluation of BCC samples (n = 24) due to strong overall staining (including epidermis) in certain samples, resulting in a Cohen’s Kappa of 0.732 (95% CI: 0.561–0.904, substantial agreement) and a Krippendorff’s alpha of 0.765 (valid for immunohistochemistry).

## 3. Discussion

In this study, selected common cutaneous tumor entities (BCC, SCC, MCN, MM) were examined with regard to the immunohistochemical expression pattern of TRPV5. BCC showed a significantly reduced frequency of positive TRPV5 staining compared to SCC. An amount of 58.3% of the BCC samples exhibited a negative staining reaction. The other tumor entities analyzed (MM dermal/epidermal, MCN epidermal) exhibited significant differences compared to BCC. Studies have been reviewed in cBioPortal to ascertain the mutation frequency of *TRPV5* across various tumor types. For MM, up to 12% mutation frequency was reported for *TRPV5* (Supplementary Figure S10). Supplementary Figure S11 depicts the mutation rate of non-melanoma skin cancer, in which BCC has substantially fewer mutations in *TRPV5* as compared to SCC. The correlation between mutation frequency and protein expression level is complex and depends on the nature of each individual mutation. Obviously, no direct conclusions can be drawn about TRPV5 expression levels based purely on data regarding the frequency of mutations.

In our work, TRPV5 protein expression was significantly lower in BCC compared to SCC. These results are similar to our previous findings regarding other pH-sensitive ion channels and G protein-coupled receptors, including TRPC5, TRPC4, GPR31, GPR151, TASK1, TASK3, and ASIC2, which also showed lower expression in BCC than in SCC. Although there is limited information on the expression of TRPV5 in skin cancers, an association between TRPV5 expression and tumor growth has been described in other tissues. In addition, TRPV5, along with other pH-sensitive membrane proteins, may represent a possible biomarker that is independent of classical clinicopathological markers for the differentiation of BCC and SCC [26].

Furthermore, a statistically significant reduction in TRPV5 mRNA expression was detected in tumor tissues of colorectal cancer compared to healthy tissue [27]. In non-small-cell lung cancer, a low expression of TRPV5 was observed and it was related to a shorter survival time following surgery [28]. Of particular note is the lower expression of TRPV5 in renal cell carcinomas. TRPV5 was expressed 38-fold lower than in healthy kidney tissue. A link with the presence of the vitamin D receptor was observed in this entity. Studies reveal that altered vitamin D receptor expression via TRPV5 channels may play a significant role in the development of renal cell carcinomas [29]. It has also been established in breast cancer that a vitamin D deficit stimulates the proliferation of human breast cancer cells in the bone. Vitamin D is a vitamin that is best evaluated via the blood level of 25-hydroxyvitamin D and has antiproliferative and cell-killing properties [30]. While TRPV5 can possibly be activated by vitamin D, its role in cancer cells, especially in renal cell carcinoma, is complex. A negative relationship between renal cell carcinomas and the expression of vitamin D receptor and TRPV5 was analyzed, indicating a possible feedback mechanism in this signaling pathway [31].

However, our findings are consistent with a tumor-type-specific expression pattern of TRPV5 in cutaneous malignancies. The lower expression in BCC, a tumor entity with a well-known extremely low metastatic potential, as compared to SCC, is consistent with our previous results on pH-sensitive membrane proteins in these types of skin cancer. Based on the available data, it is not possible to determine with certainty whether this difference in gene expression is biologically linked to the differing metastatic behavior of these entities. The consistent finding across several independent studies by our group that BCC exhibit lower expression of pH-sensitive membrane proteins—including TRPC4, TRPC5, GPR31, GPR151, TASK1, TASK3 and ASIC2—lends biological plausibility to the present findings and suggests that the tumor microenvironment in basal cell carcinomas may differ fundamentally from that in SCC at the level of pH-sensitive signal transduction. Despite the fact that our results contrast with the expression pattern in other tumors, they very well do match all other findings regarding pH-sensitive proteins in the investigated skin cancers. In summary, it can be concluded that TRPV5 could potentially be useful as a diagnostic or prognostic biomarker for skin cancer. However, further functional studies are needed to fully understand its biological relevance and to determine whether, beyond differences in expression levels, it contributes to tumor behavior.

Given the sample size of this first study on the expression of TRPV5 in skin tumors, correlations with more clinical parameters would not allow for meaningful analyses. Digital quantification tools such as the IHC Profiler perform pixel-level analysis of DAB-stained IHC images and show a very high degree of agreement with manual pathological analysis in standardized tissue samples [32]. Its reliability with old paraffin-embedded archive material, as is the case in this study, cannot be guaranteed. However, in future studies, digital image analysis in addition to the evaluation by experienced dermatopathologists might be a valuable means to improve the methodological spectrum used to analyze expression patterns.

## 4. Materials and Methods

### 4.1. Tissue Samples

Paraffin-embedded tissue samples obtained from the dermatopathological routine laboratory at the Department of Dermatology, University Medical Center Regensburg, were used in the experimental procedures. Only samples older than 10 years were selected for this study. Pursuant to German legislation, after the expiry of the statutory maintenance period of 10 years, these archived and anonymized samples are considered to be available for utilization in scientific research purposes. Otherwise, the respective samples would have been discarded routinely. The information provided does not allow for patient identification.

### 4.2. Immunohistochemistry

The tissue was fixed in formalin and embedded in paraffin. Subsequently, individual sections of 3 µm were cut with the aid of a microtome and mounted on microscope slides. To identify the tumor area, each sample was first stained with hematoxylin and eosin (HE). Paraffin was eliminated from the slides, and the tissue was blocked by incubation for 30 min at 75 °C. Next, the slides were rehydrated using a gradient of alcohol concentrations as follows: 3× xylol for 10 min, 2× 100% ethanol for 10 min, 2× 96% ethanol for 10 min, and 2× 70% ethanol for 10 min. In order to inhibit endogenous peroxidase activity, tissue sections were immersed in 3% H_2_O_2_ for 10 min. An concentration of 3% H_2_O_2_ was generated by combining 3 mL of 30% H_2_O_2_ (Roth, Karlsruhe, Germany, No. 8070.1) with 100 mL of 70% ethanol. Meanwhile, citrate buffer at pH 6 (Zytomed, Berlin, Germany, ZUC028-500) was heated for 30 min. The slides were rinsed with distilled water and then boiled for 20 min in preheated citrate buffer. After this procedure, they were cooled on ice for 20 min. Afterwards, they were transferred to Phosphate-Buffered Solution (PBS) (Sigma-Aldrich, Darmstadt, Germany, No. D8537) and incubated for 10 min via gentle shaking. Following that, protein blocking solution (Zytomed, Berlin, Germany, ZUC007-100) was applied to the slides for 5 min, then they were transferred to washing buffer solution (Zytomed, Berlin, Germany, ZUC020-500) for a further 5 min under gentle shaking. The tissue samples were treated overnight at 4 °C with the primary rabbit anti-human TRPV5 polyclonal antibody (Alomone Labs, Jerusalem, Israel, 0.8 mg/mL, RRID: AB_2039793). The antibody was diluted 1:200 with Antibody Diluent (Zytomed, Berlin, Germany, REF ZUC025-500) prior to application on the slides. The primary antibody was incubated at 4 °C overnight. They were rinsed three times with washing buffer solution on the following day prior to incubating the tissue sections with secondary antibody (Nichirei, Histofine simple stain rabbit, Tokyo, Japan, No. 414141F) for 30 min. The subsequent step was to apply the chromogen solution (AEC) to the tissue sections to render the staining process visible. The AEC substrate must be freshly produced for each staining procedure due to its instability. Consequently, we incorporated 20 μL of AEC chromogen into 10 mL of AEC Substrate Buffer and mixed the solution (Zytomed, Berlin, Germany, No. ZUC42-500).

After reaching the required degree of coloration, the reaction was stopped with distilled water. Counterstaining of the nuclei was performed with Mayer’s Hemalaun for 1–2 min (Roth, Karlsruhe, Germany, No. T865.3) and “blued” with tap water. We scanned the stained sections with the PreciPoint M8 and processed the digital images with ViewPoint Online (PreciPoint, M8 system, Garching bei München, Germany).

### 4.3. Western Blots

A Western blot (WB) analysis was done to verify the specificity of the TRPV5 antibody. Cells were initially taken from 75 cm^2^ cell culture flasks at approximately 70–80% confluence using trypsinization for the WB procedure. The resultant cell pellets were rinsed with PBS and subsequently resuspended in a self-prepared RIPA buffer for protein extraction. The protein concentration was quantified via a BCA protein assay (Pierce BCA Kit, Thermo Scientific, Waltham, MA, USA). All samples were then adjusted to a protein content of 2 µg/µL. The samples were combined with Laemmli Sample Buffer and DTT in a 4:1 ratio and subjected to heating at 90 °C for 10 min for denaturation. The proteins were fractionated using SDS-PAGE on a Mini-PROTEAN TGX Stain-Free Gel (Any kD, Bio-Rad, Hercules, CA, USA). An amount of 20 µg (=10 µL) of the denatured material was deposited per lane. Electrophoresis was conducted at a voltage of 200 V for 35 min. Tris/Glycine/SDS (10×, diluted 1:10 in distilled water) served as a running buffer. Next, proteins were transferred to a PVDF membrane (Bio-Rad, Hercules, CA, USA) using wet blotting. The transfer was conducted at 150 mA for 1 h in a buffer of 100 mL of 10× Tris/Glycine Buffer, 200 mL of 100% methanol, and adjusted to 1 liter with distilled water. The membrane was incubated for 2 h at room temperature in 10% goat serum in PBST with constant agitation to block non-specific antibody binding sites. The primary antibody against TRPV5 (Anti-TRPV5, Alomone Labs, Jerusalem, Israel, 1:500 in 5% goat serum/PBST) was incubated overnight at 4 °C with continuous shaking to ensure homogeneous mixing. Following several washing cycles with PBST, the blot was incubated with the secondary fluorescence-labeled antibody (StarBright 700 Goat Anti-Rabbit, Bio-Rad, Hercules, CA, USA, 1:10,000 in PBST) for one hour at room temperature, always under constant agitation on a shaker. Subsequent washing processes, culminating in pure PBS, allowed for the analysis and visualization of the signals utilizing the ChemiDoc Imaging System from Bio-Rad. Protein separation was monitored using Unstained Protein Standard (10–250 kDa; New England Biolabs, Ipswich, MA, USA; cat. no. P7717S) and Precision Plus Protein All Blue Prestained Protein Standards (10–250 kDa; Bio-Rad Laboratories, Hercules, CA, USA; cat. no. 161-0373).

### 4.4. Scoring

Dermatologists with extensive experience in dermatopathology from the Department of Dermatology at the University Medical Center in Regensburg performed visual assessments of H&E and TRPV5 staining. Each sample was independently evaluated by two experts without prior knowledge of the other rater’s assessment. The ranking represents a semi-quantitative evaluation, i.e., ranks on an ordinal scale. Unaffected portions of the epidermis of each individual sample served as an internal reference for each slide, thereby eliminating inter-sample staining variability as a confounding factor. Staining was classified into the following categories: ++ for strong positive responses (over 80% of cells either positive or exhibiting intense staining), + for weak-positive or partially positive responses (20–80% of cells displaying weak or partially strong staining), and − for negative responses (fewer than 20% of cells showing weak staining). A slight tint in some images may be due to suboptimal white balance that does not affect the overall assessment because unaffected portions of the epidermis served as internal reference for individual samples. As stated above, this also served to prevent confounding by inter-sample variability due to overall staining differences in individual samples.

### 4.5. Statistics

In our dataset, the rating values originate from a semi-quantitative evaluation (ordinal scale) as they can be sorted/ranked in a specific order. The Kruskal–Wallis test was used to check for differences in the scoring results between the 4 analyzed tumor entities. Epidermal and dermal portions of MCN and MM were checked separately. This was not done for BCC and SCC, as both are epithelial tumors that originate from the epidermis and are connected with it. Pairwise comparisons were conducted using the Bonferroni test. Only comparisons with an adjusted p-value of less than 0.05 following Bonferroni correction were considered significant. To check for inter-rater reliability, we used Cohen’s Kappa with quadratic weights and Krippendorff’s alpha for ordinal-scale data.

## 5. Conclusions

This study is the first one on the expression of the pH-sensitive protein TRPV5 in skin tumors. While our methodology is mainly descriptive, it introduces TRPV5 as a potential novel immunohistochemical marker, which may help support the differentiation between BCC and SCC based on the result that TRPV5 expression was significantly lower in BCC as compared to SCC. Nonetheless, our findings require analysis in larger studies. In addition, adnexal tumors should also be considered in further studies, as they play an important role in the differential diagnosis of epithelial tumors. The main limitation of this study is its descriptive nature, which is primarily due to the fact that this is the first study on the expression of TRPV5 in skin tumors. Based on our results, functional data also need to be added in future studies. The role of TRPV5 in proliferation, migration, and cell survival could be clarified by using cell lines with knockout and overexpression of TRPV5 exposed to varied pHe in the culture media. Following the identification of individual protein levels in diverse cell types utilizing qPCR and Western blot, knockdown (siRNA)/knockout (CRISPR/Cas9) and overexpression approaches should be applied alongside functional cellular assays as the next step to study the topic, as we proposed earlier [22].

## Figures and Tables

**Table 1:** Results of statistical analysis.

Pairs	*p*-Value	Adj. *p*-Value
BCC-MCN dermal	0.006	0.097
BCC-MM dermal	<0.001	<0.001
BCC-MCN epidermal	<0.001	<0.001
BCC-SCC	<0.001	<0.001
BCC-MM epidermal	<0.001	<0.001
MCN dermal-MM dermal	0.136	1.000
MCN dermal-MCN epidermal	0.089	1.000
MCN dermal-SCC	0.080	1.000
MCN dermal-MM epidermal	0.015	0.226
MM dermal-MCN epidermal	0.787	1.000
MM dermal-SCC	0.772	1.000
MM dermal-MM epidermal	0.328	1.000
MCN epidermal-SCC	0.990	1.000
MCN epidermal-MM epidermal	0.501	1.000
SCC-MM epidermal	0.499	1.000

Green: highlights the relevant results.

## Data Availability

Raw data are available in the Supplementary Information and upon reasonable request.

## Supplementary Materials

The following supporting information can be downloaded at: https://www.mdpi.com/article/10.3390/ijms27125287/s1.
