# Supplementary material for: Immunohistochemical Analysis of pH-Sensitive TRPV5 in Common Skin Tumors
Source: Int J Mol Sci. 2026 Jun 11;27(12):5287. doi: 10.3390/ijms27125287 (PMC13299883; doi:10.3390/ijms27125287)
Supplement: Supplementary file 1 [file ijms-27-05287-s001.zip › ijms-4277434-supplementary.pdf]

SUPPLEMENTARY INFORMATION

Supplementary Figure S1: Western Blot of TRPV5.

A

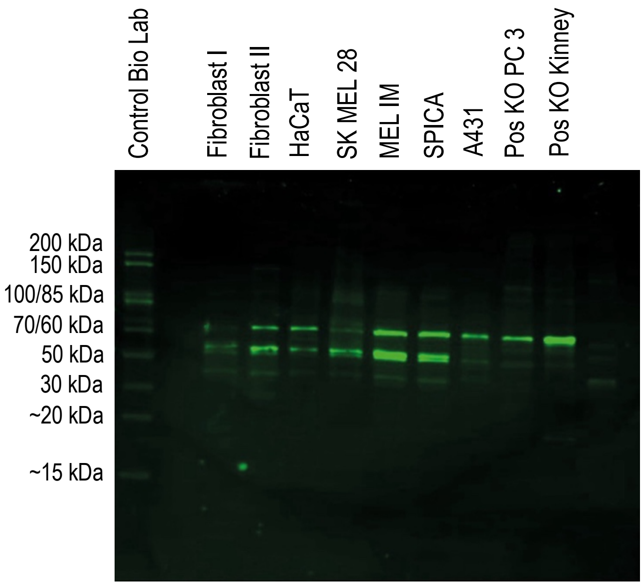

B

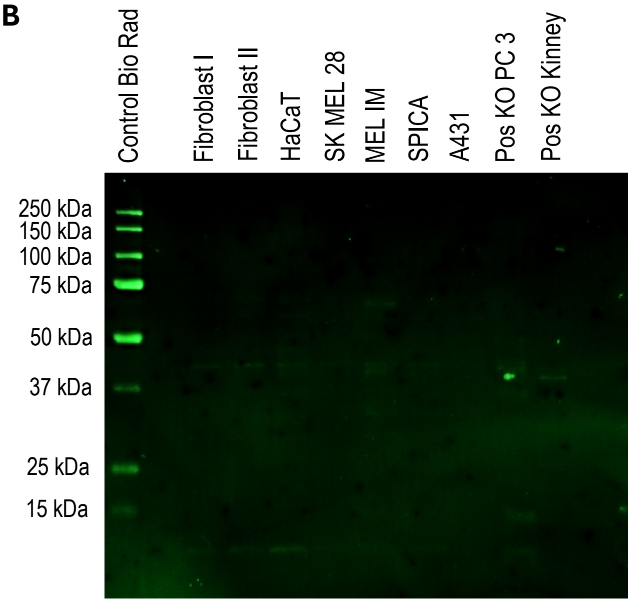

A: Original Version of Figure 2B Western Blot. Assessment of the specificity of the TRPV5 antibody via western blot at a dose of 20 µg (10 µL) per lane in different cell lines in comparison to the best-suited Marker Bio Lab (see methods). Gel: any kDa TGX Precast stain-free Gel Primary AB: Anti TRPV 5 1:750; 2 bands at approximately 70 kDa and 60 kDa. Secondary AB: StarBright 700 Fa. BioRad. Normalization was conducted based on the total protein content of each lane, rather than utilizing a specific housekeeping protein, as facilitated by the TGX stain-free gel system. Lanes 2 and 12 are empty.

B: Original Version of Figure D Western Blot. To verify the specificity of the anti-TRPV5 antibody, preincubation with the corresponding blocking peptide was conducted, which resulted in an almost complete loss of signal in all lanes except for the the secondary standard control lane (Bio Rad, see methods) without blocking peptide. Lanes 2 and 12 are empty.

Supplementary Figures S2-3: Immunohistochemistry for TRPV5 in BCC.

Scale bars represent 200 µm. In BCC, TRPV5 reaction was weak positive for 9/24 samples (1, 3, 4, 5, 6, 8, 10, 11, 18). 14/24 (7, 9, 12, 13, 14, 15, 16, 17, 19, 20, 21, 22, 23, 24) showed a negative staining and 1/24 showed a strong positive expression (2).

Supplementary Figure S2: Immunohistochemistry for TRPV5 in BCC (part 1)

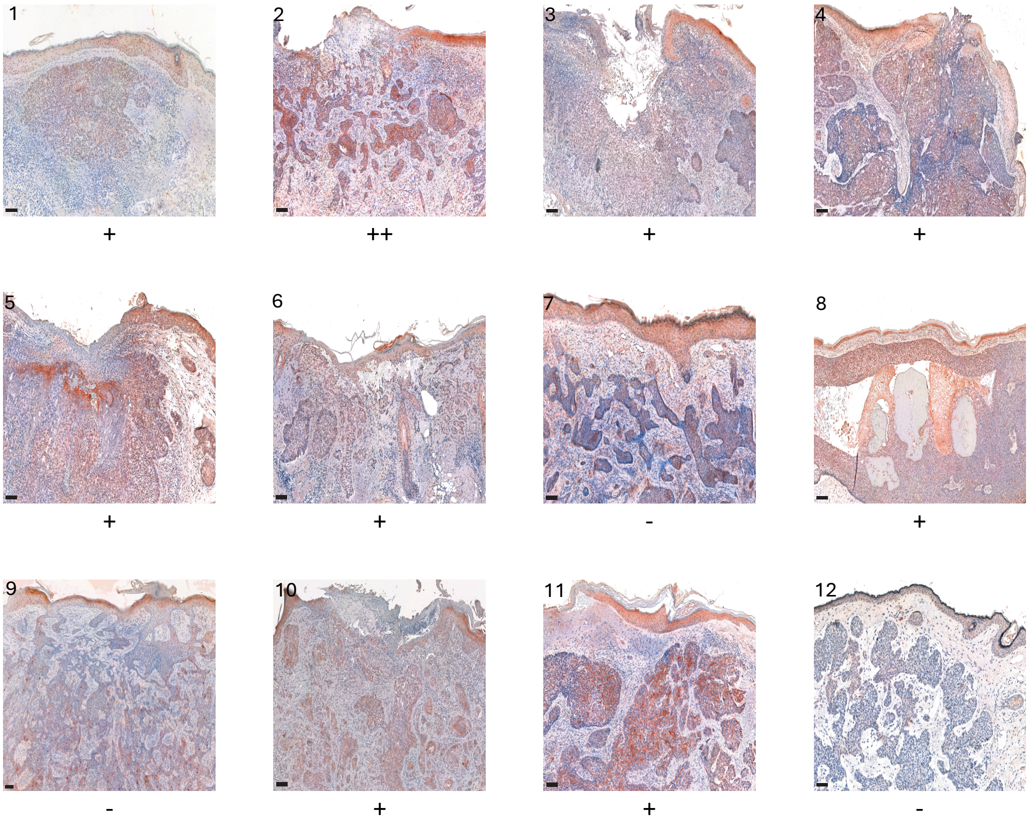

Supplementary Figure S3: Immunohistochemistry for TRPV5 in BCC (part 2)

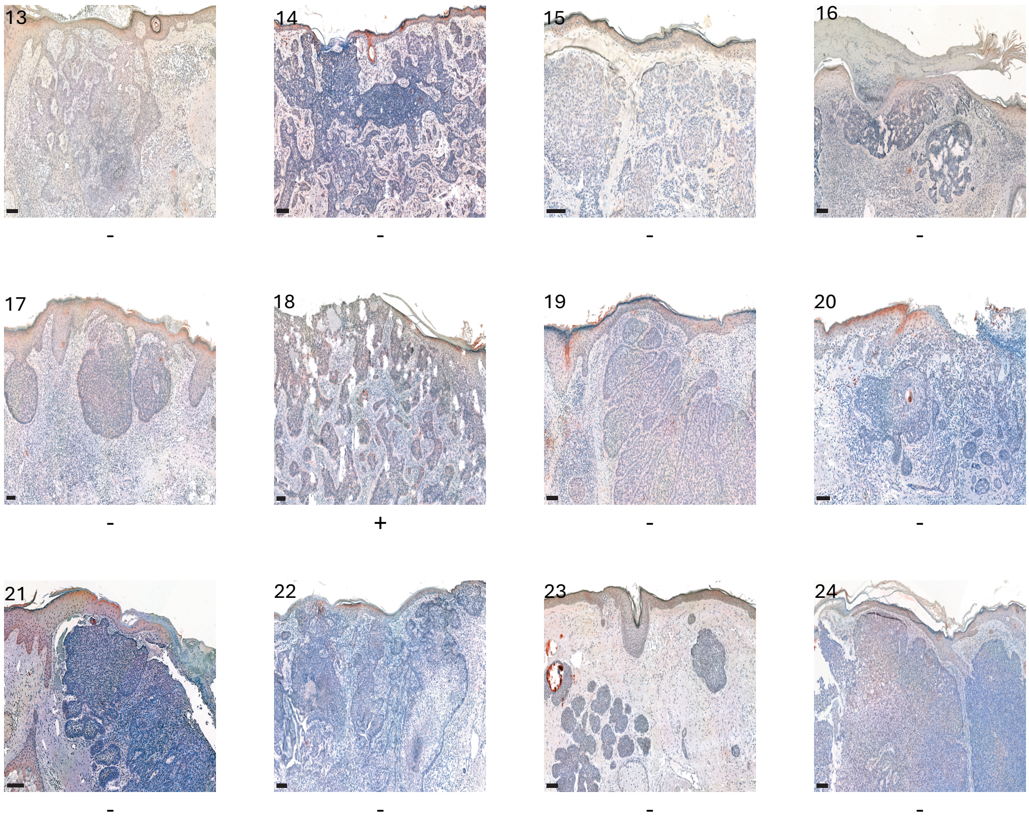

Supplementary Figures S4-5: Immunohistochemistry for TRPV5 in SCC.

Scale bar represents 200 µm. All 21 SCC samples (1-21) showed a pretty homogenous expression, representing weak positive 11 / 21 (1, 6, 7, 8, 12, 13, 14, 16, 17, 20, 21) and strong positive expression 10/21 (2, 3, 4, 5, 9, 10, 11, 15, 18, 19).

Supplementary Figure S4: Immunohistochemistry for TRPV5 in SCC (part 1)

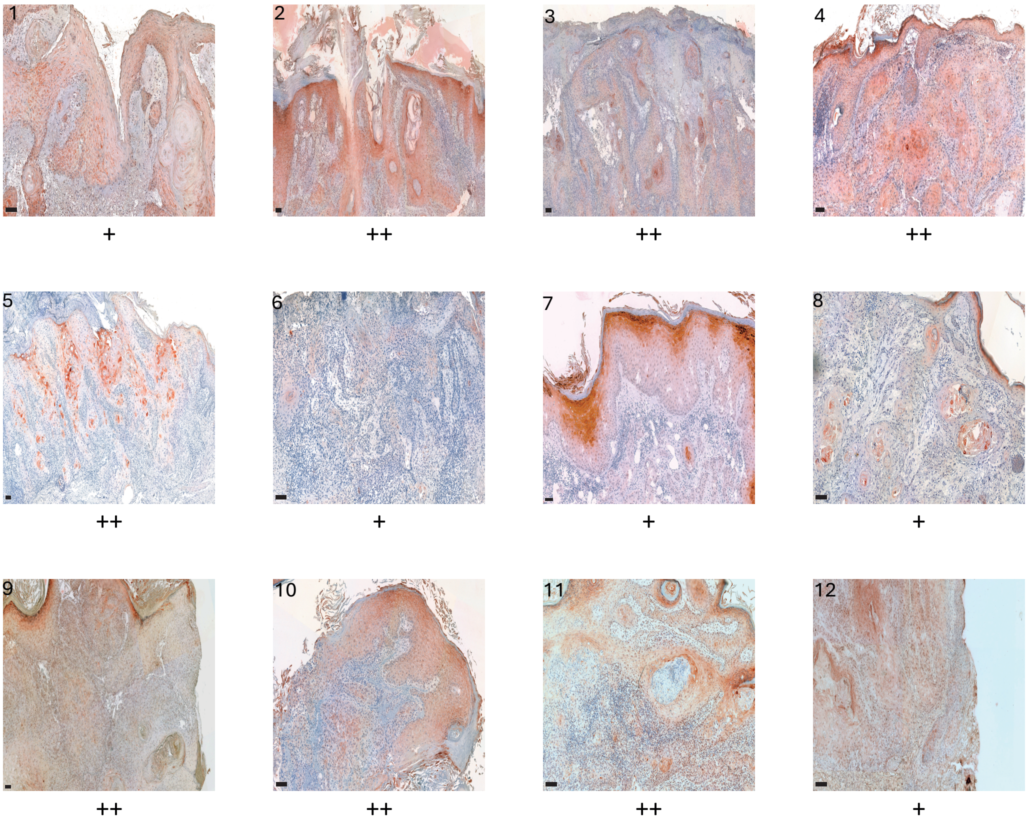

Supplementary Figure S5: Immunohistochemistry for TRPV5 in SCC (part 2)

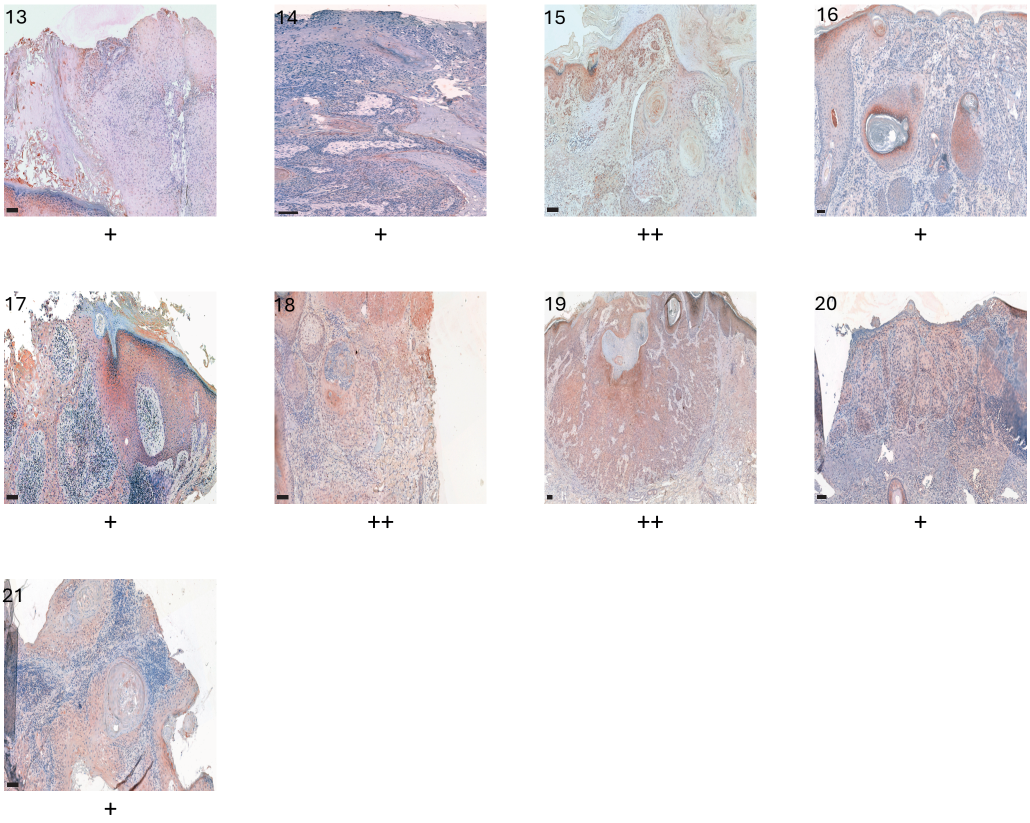

Supplementary Figures S6-7: Immunohistochemistry for TRPV5 in MCN.

Scale bar represents 200 µm. The epidermal portion, 9/19 showed a strong positive reaction for TRPV5 (1, 5, 6, 7, 8, 10, 11, 16, 17), 10/19 reacted weakly positive (2, 4, 9, 12, 13, 14, 15, 18, 19, 20). In 13/19, TRPV5 represents a weak positive expression in the dermal portion (1, 2, 3, 4, 6, 9, 10, 11, 12, 15, 16, 18, 20) and number 13 and 14 showed a negative staining.

Supplementary Figure S6: Immunohistochemistry for TRPV5 in MCN (part 1)

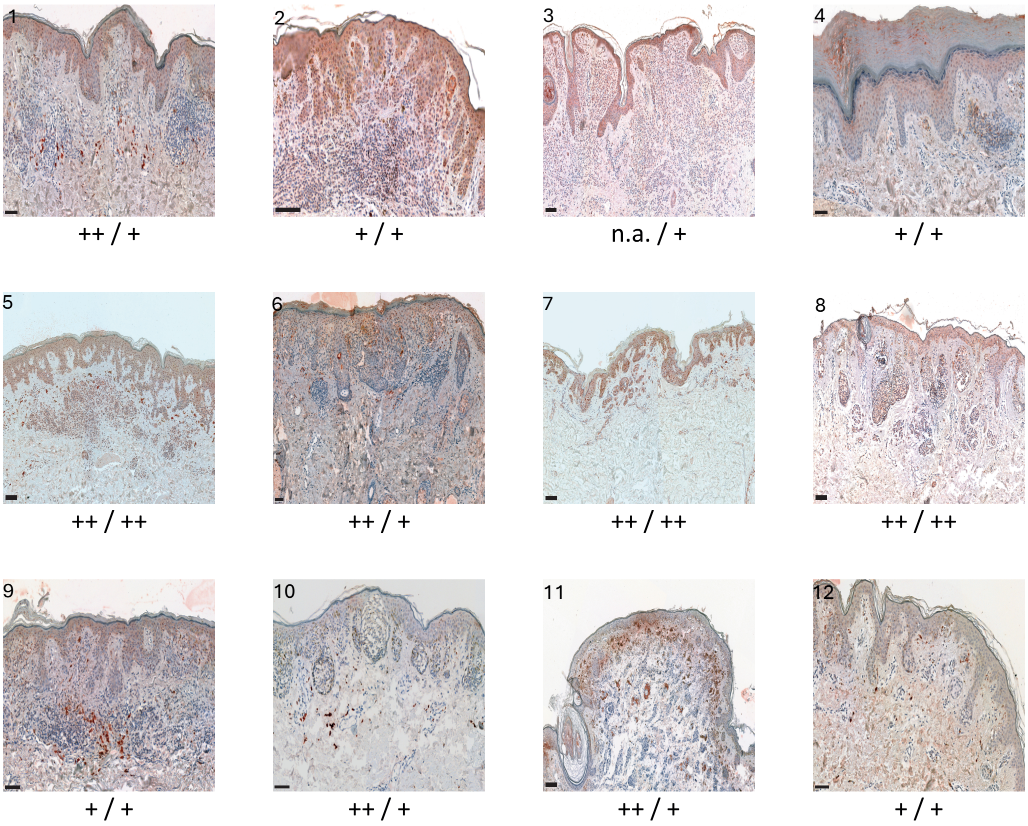

Supplementary Figure S7: Immunohistochemistry for TRPV5 in MCN (part 2)

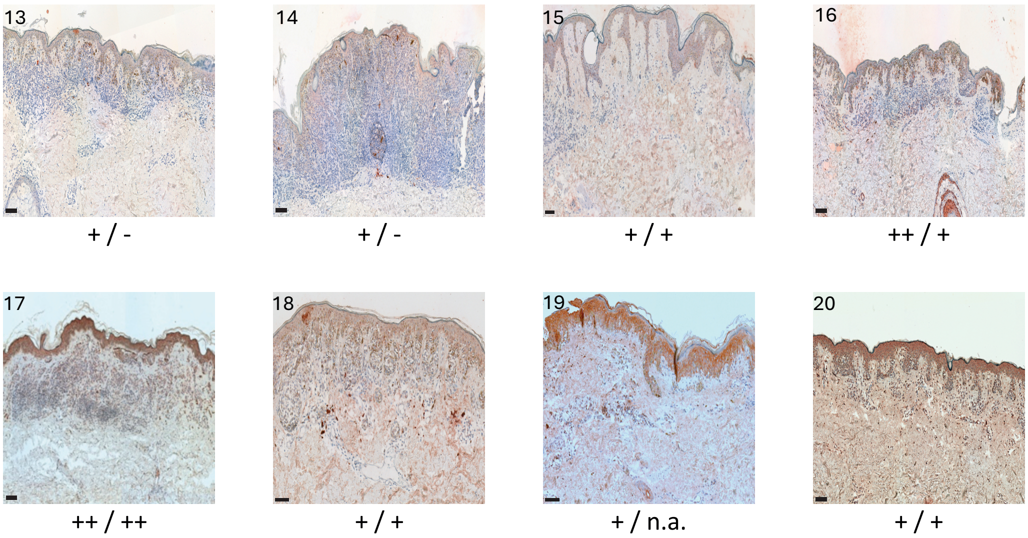

Supplementary Figures S8-9: Immunohistochemistry for TRPV5 in MM.

Scale bar represents 200 µm. 10/22 of the epidermal and dermal sections were both strong positive for TRPV5 (1, 3, 4, 6, 11, 13, 15, 16, 21, 22). Only strong positive in the epidermal parts and weak positive in the dermal parts were 4/22 (2, 5, 10, 12). For 6/22 samples both reactions were displaying weakly positive (7, 8, 9, 14, 17, 19) whereas number 18 and 20 represent a weakly positive and negative staining reaction.

Supplementary Figure S8: Immunohistochemistry for TRPV5 in MM (part 1)

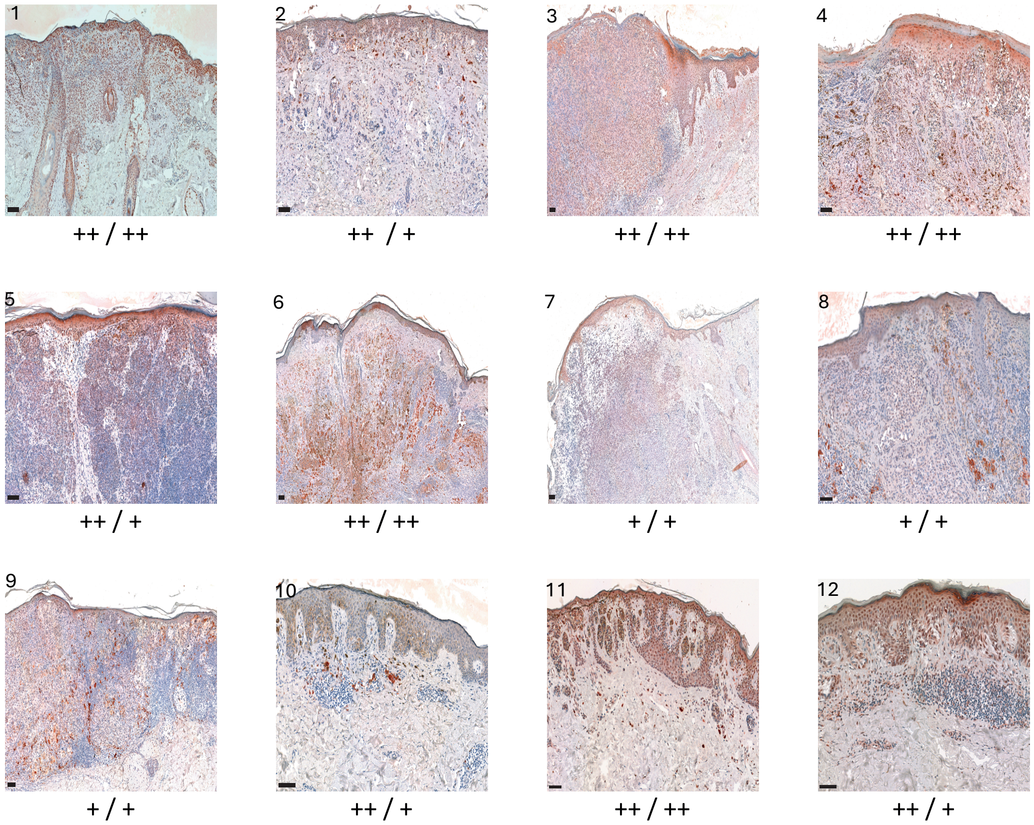

Supplementary Figure S9: Immunohistochemistry for TRPV5 in MM (part 2)

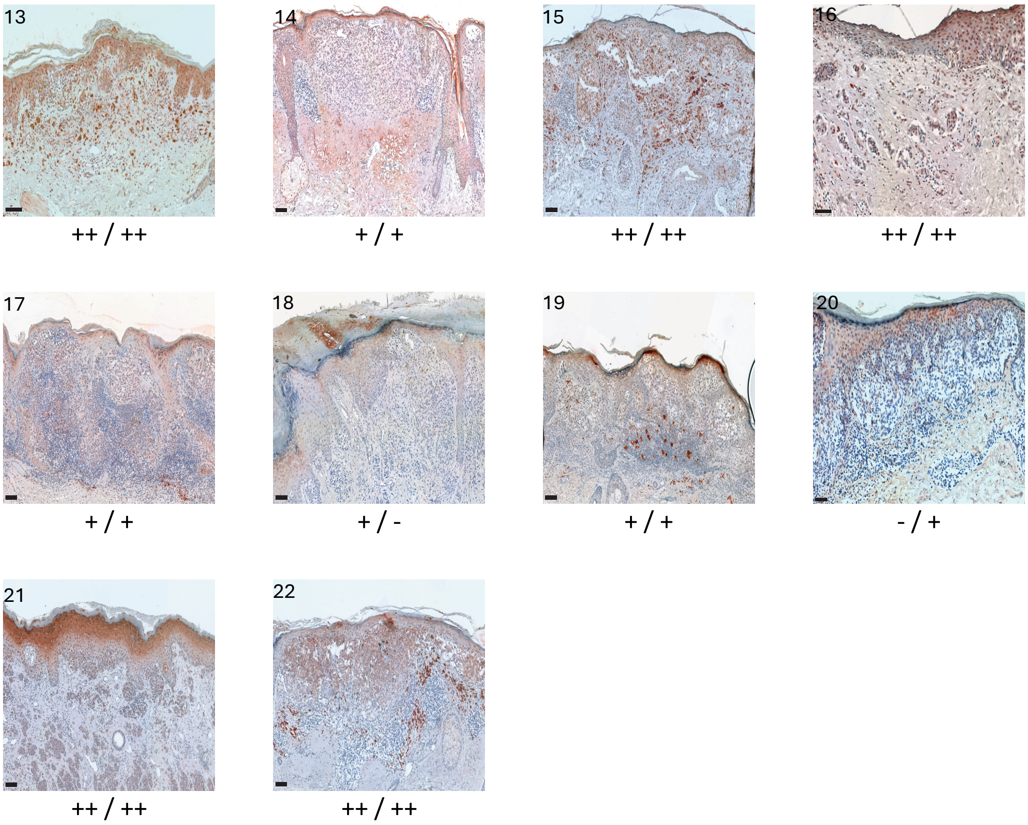

Supplementary Figure S10:TRPV 5 mutation frequency in MM from cBioportal.org

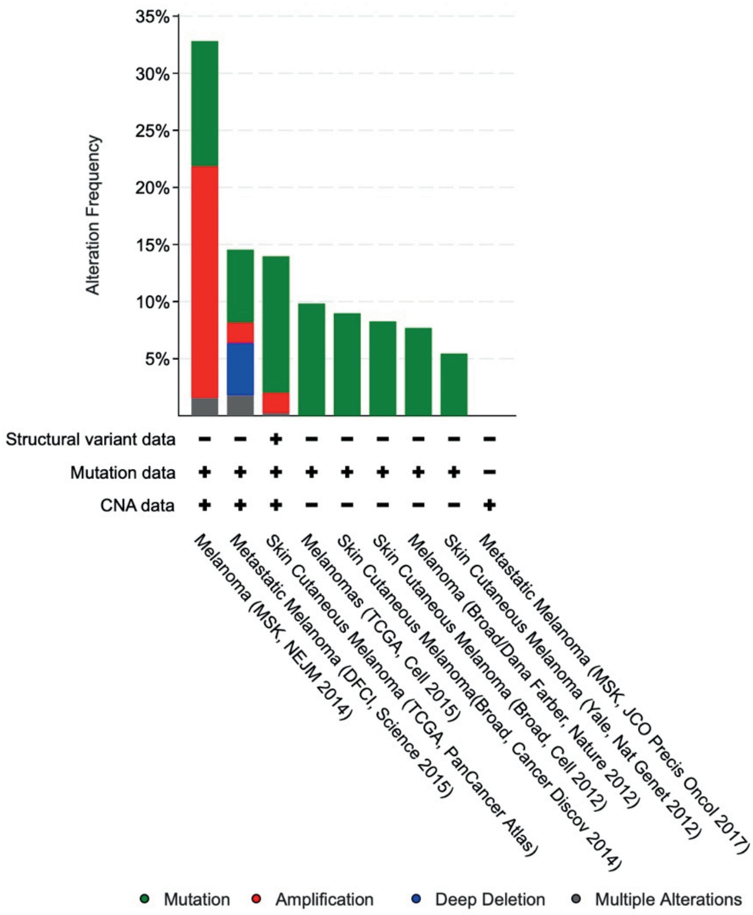

Supplementary Figure S11: TRPV 5 mutation frequency in non-melanoma skin cancer from cBioportal.org

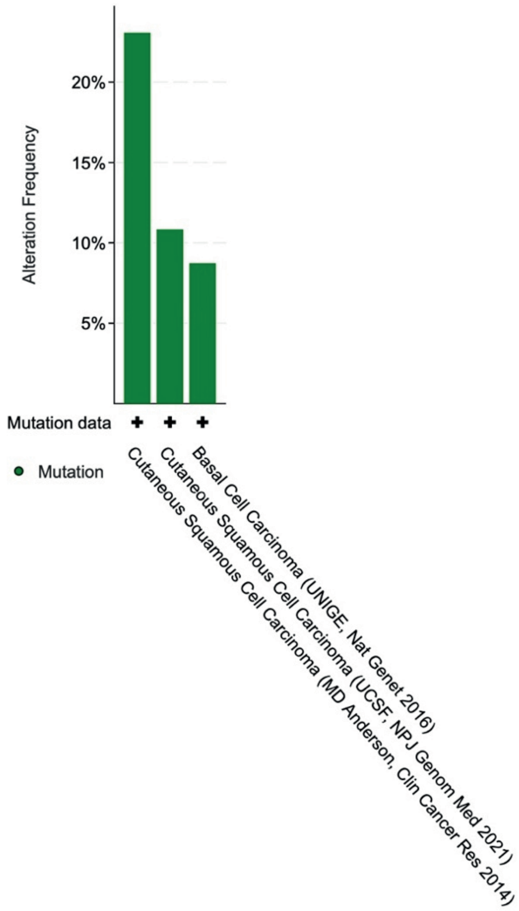

Supplementary Table S1: Scoring results for TRPV 5 in BCC

Number	Sex	Age	Type of BCC	Scoring
1	m	75	nodular	+
2	m	68	nodular/sclerosing	++
3	m	49	nodular	+
4	m	56	nodular	+
5	m	59	nodular	+
6	f	88	nodular	+
7	m	85	nodular	-
8	m	75	nodular	+
9	m	41	nodular	-
10	m	36	nodular	+
11	f	85	nodular	+
12	f	79	nodular	-
13	m	74	nodular	-
14	f	84	nodular	-
15	f	51	nodular	-
16	m	73	superficial	-
17	m	85	superficial	-
18	m	82	nodular	+
19	m	63	nodular	-
20	f	74	nodular	-
21	m	57	nodular	-
22	f	65	nodular	-
23	m	68	nodular	-
24	m	72	nodular	-

Supplementary Table S2: Scoring results for TRPV 5 in SCC

Number	Sex	Age	Scoring
1	f	82	+
2	f	74	++
3	m	72	++
4	f	87	++
5	m	74	++
6	m	63	+
7	m	65	+
8	f	71	+
9	m	65	++
10	m	46	++
11	m	75	++
12	f	63	+
13	m	47	+
14	m	63	+
15	m	78	++
16	m	90	+
17	m	91	+
18	m	73	++
19	f	73	++
20	m	96	+
21	f	89	+

Supplementary Table S3: Scoring results for TRPV 5 in MCN

Number	Sex	Age	Type of MCN	Scoring Epidermal	Scoring Dermal
1	f	23	compound	++	+
2	f	40	compound	+	+
3	f	42	dermal	NA	+
4	m	21	compound	+	+
5	m	41	compound	++	++
6	m	44	compound	++	+
7	m	37	compound	++	++
8	m	24	compound	++	++
9	f	50	compound	+	+
10	m	33	compound	++	+
11	f	32	compound	++	+
12	f	38	compound	+	+
13	f	37	compound	+	-
14	m	15	compound	+	-
15	f	27	compound	+	+
16	m	47	compound	++	+
17	f	37	compound	++	++
18	m	33	compound	+	+
19	f	46	junctional	+	NA
20	f	51	compound	+	+

Supplementary Table S4: Scoring results for TRPV 5 in MM

Number	Sex	Age	Scoring Epidermal	Scoring Dermal
1	f	33	++	++
2	f	30	++	+
3	f	82	++	++
4	f	83	++	++
5	f	83	++	+
6	f	42	++	++
7	m	59	+	+
8	f	47	+	+
9	m	77	+	+
10	m	50	++	+
11	m	56	++	++
12	f	51	++	+
13	f	71	++	++
14	f	69	+	+
15	m	56	++	++
16	m	63	++	++
17	f	62	+	+
18	f	111	+	-
19	f	43	+	+
20	f	45	-	+
21	f	75	++	++
22	m	81	++	++

Supplementary Table S5: Statistical analysis of TRPV 5 – comparison of all entities

		Scoring			Total
		-	+	++	
**MCN epidermal**	number	0	10	9	19
	% of tumors	0.0%	52.6%	47.4%	100.0%
**MCN dermal**	number	2	13	4	19
	% of tumors	10.5%	68.4%	21.1%	100.0%
**MM epidermal**	number	1	7	14	22
	% of tumors	4.5%	31.8%	63.6%	100.0%
**MM dermal**	number	1	11	10	22
	% of tumors	4.5%	50.0%	45.5%	100.0%
**SCC**	number	0	11	10	21
	% of tumors	0.0%	52.4%	47.6%	100.0%
**BCC**	number	14	9	1	24
	% of tumors	58.3%	37.5%	4.2%	100.0%
**Total**	number	18	61	48	127
	% of tumors	14.2%	48.0%	37.8%	100.0%
